# Are Seals Skinned Alive?

**Published:** 1910-01-08

**Authors:** 


*To the Editor of* THE HOSPITAL.

### ARE SEALS SKINNED ALIVE?

*To the Editor of* THE HOSPITAL.

SIR,—I have been reading a review of Mr. Joseph Collinson's booklet, entitled "The Fate of the Fur Seal," written by Sir Herbert Maxwell, who is so well able to deal with the subject. May I be allowed to call the attention of your readers to the following passage which occurs in Sir Herbert's interesting article?

"I write of what I have seen over and over again without being able to prevent it, for the excitement and the sight of the blood seems to turn our fellows into fiends incarnate for the time being. Take this one day's sealing as an example. The ice was strewn thickly with baby seals; and not even a lamb itself is more lovely or innocent-looking than one of those. They are about two or two-and-a-half feet long, and swaddled, as it were, from head to tail with skin covered with long yellow-white hair. Barring the wee black nose and the jet black tender loving eyes, there is hardly another feature distinguishable, so well has Nature wrapped them up against the cold. They never attempt to move off—they cannot. One blow from the sharp end of the club and the baby is weltering in its gore. The skinning takes place immediately, the blubber and skin being removed together, and often pieces of the dark and quivering flesh. The killing of the young creature before flensing is humane enough. But this is not always done. Oftentimes the baby is only partially stunned, and when flayed may be seen to roll in agony on the snow. But beasts in shape of human beings at times skin them alive! And I have seen these fellows pitch a living flayed seal into the water to see whether it would move off or not. . . ."

So that the fur-seal trade is, truly enough, the story of the egret over again, different in form, but as full of blood-curdling horrors, if not worse. Is there a remedy, and can it be applied? I have heard various suggestions discussed; but at best they are clumsy palliatives. There is, however, one certain and effective way of stopping all the cruel and brutal practices which are bringing about the extermination of the fur seal, and that is for women to renounce those articles of clothing which can only be obtained at the cost of such agony to a harmless and beautiful animal. In conclusion, may I refer those of your readers who are interested in the fur-seal question to the Hon. Secretary of the Humanitarian League, 53 Chancery Lane, London, for fuller information and literature?

I am, yours faithfully,
HUMANITY.

